# Alterations in Striatal Architecture and Biochemical Markers’ Levels During Postnatal Development in the Rat Model of an Attention Deficit/Hyperactivity Disorder (ADHD)

**DOI:** 10.3390/ijms252413652

**Published:** 2024-12-20

**Authors:** Ewelina Bogdańska-Chomczyk, Paweł Wojtacha, Meng-Li Tsai, Andrew Chih Wei Huang, Anna Kozłowska

**Affiliations:** 1 Department of Human Physiology and Pathophysiology, School of Medicine, Collegium Medicum, University of Warmia and Mazury, Warszawska 30, 10-082 Olsztyn, Poland; kozlowska.anna@uwm.edu.pl; 2 Department of Psychology and Sociology of Health and Public Health, University of Warmia and Mazury, Warszawska 30, 10-082 Olsztyn, Poland; pawel.wojtacha@uwm.edu.pl; 3 Department of Biomechatronic Engineering, National Ilan University, Ylan 26047, Taiwan; mengli0320@gmail.com; 4 Department of Psychology, Fo Guang University, Ylan 26247, Taiwan; chweihuang@mail.fgu.edu.tw

**Keywords:** rat, ADHD, striatum abnormalities, SHR, morphometry, biochemical markers

## Abstract

Attention deficit/hyperactivity disorder (ADHD) is defined as a neurodevelopmental condition. The precise underlying mechanisms remain incompletely elucidated. A body of research suggests disruptions in both the cellular architecture and neuronal function within the brain regions of individuals with ADHD, coupled with disturbances in the biochemical parameters. This study seeks to evaluate the morphological characteristics with a volume measurement of the striatal regions and a neuron density assessment within the studied areas across different developmental stages in Spontaneously Hypertensive Rats (SHRs) and Wistar Kyoto Rats (WKYs). Furthermore, the investigation aims to scrutinize the levels and activities of specific markers related to immune function, oxidative stress, and metabolism within the striatum of juvenile and maturing SHRs compared to WKYs. The findings reveal that the most pronounced reductions in striatal volume occur during the juvenile stage in SHRs, alongside alterations in neuronal density within these brain regions compared to WKYs. Additionally, SHRs exhibit heightened levels and activities of various markers, including RAC-alpha serine/threonine-protein kinase (AKT-1), glucocorticoid receptor (GCsRβ), malondialdehyde (MDA), sulfhydryl groups (-SH), glucose (G), iron (Fe), lactate dehydrogenase (LDH). alanine transaminase (ALT), and aspartate transaminase (AST). In summary, notable changes in striatal morphology and elevated levels of inflammatory, oxidative, and metabolic markers within the striatum may be linked to the disrupted brain development and maturation observed in ADHD.

## 1. Introduction

Attention deficit hyperactivity disorder (ADHD) is one of the most prevalent neurodevelopmental disorders that predominantly emerges in childhood, although it often persists into adolescence and adulthood [1]. It is characterized by developmentally inappropriate enduring inattention, impulsivity, and hyperactivity patterns. These symptoms significantly disrupt daily functioning, academic achievement, occupational performance, and social interactions [2]. It has been found that ADHD is prevalent in about 11.4% of children and 6.76% of adults [3,4]. Moreover, the condition is twice as common in males as in females [5]. It is one of the most heritable mental disorders. Furthermore, smoking during pregnancy, nutritional deficiencies, fetal alcohol exposure, and viral infections have also been recognized as causes of ADHD [6].

Ultimately, the exact pathogenesis of disorder has not been sufficiently understood. Neuroimaging studies have revealed structural and functional abnormalities in specific brain regions of individuals with ADHD. For example, Almeida et al. [7] have shown a reduced right frontal cortical thickness in children, adolescents, and adults with this disorder. In addition, the cohort lifetime study has demonstrated reduced fronto-striatal volumes in ADHD children patients [8]. Several studies using volumetric brain imaging techniques have highlighted the significant involvement of the basal ganglia (BG) in the underlying mechanisms of ADHD [9,10,11,12,13,14,15,16]. The BG is a group of subcortical nuclei (clusters of neurons), including the two output nuclei: the globus pallidus internal segment, substantia nigra pars reticulata, and the main input nucleus—the striatum (CPu; caudate putamen) [17]. The BG receives input from the neocortex via the striatum, where the sensory and cognitive information is processed and integrated. Next, the BG relay those signals to the prefrontal cortex (PFC), which is responsible for motor planning, coordination, cognitive control, and the ability to adapt and learn new movements [18]. It should be noted that abnormalities in the striatum are closely associated with ADHD symptoms, affecting key areas of behavior and cognitive functions. Specifically, concentration problems in ADHD may stem from deficits in the role of the striatum in the reward system, leading to difficulties in maintaining attention, particularly during tasks that do not provide immediate satisfaction [19,20]. Furthermore, hyperactivity and impulsivity in ADHD are also linked to striatal dysfunction, which is crucial for motor control, planning, and inhibiting inappropriate behaviors. Individuals with ADHD may struggle with executive functions, such as cognitive flexibility and planning, due to disrupted connections between the striatum and the PFC [21]. The literature indicates that, neurobiologically, ADHD is associated with dopaminergic dysfunction in the striatum, which plays a key role in motivation and attention. Notably, the study by Castellanos et al. [22], employing a magnetic resonance imaging (MRI) investigation on monozygotic twins discordant for ADHD, unveiled a substantial reduction in the volume of the CPu. Additionally, a separate neuroimaging study involving school-aged girls with ADHD demonstrated a correlation between diminished volumes of the pallidum and CPu and the severity of ADHD symptoms and cognitive performance [23]. In addition, it have been shown that the ventral striatum exhibits a smaller volume [24,25] and lower activation during reward anticipation in ADHD individuals [19]. Moreover, several studies have reported altered functional connectivity, particularly in the caudate nucleus and putamen, further linking striatal abnormalities to the core symptoms of ADHD [26,27,28]. Interestingly, researchers suggested that the volume changes in the CPu seen in children with ADHD might not be permanent but temporary [10]. Notably, these differences were more pronounced in younger children, gradually diminishing as the individuals approached adolescence [10]. Intriguingly, our team’s prior investigations, conducted on the validated Spontaneously Hypertensive Rat (SHR) model of ADHD, yielded insights into volume alterations in movement-related regions among juvenile SHRs. These alterations encompassed reductions in the PFC [29], primary motor cortex (M1), and secondary motor cortex (M2) [30], and a decrease in the total striatal volume [31].

Moreover, the current literature underscores that ADHD is recognized as a multifaceted disorder, attributed to the intricate interplay of various interconnected factors and dimensions [32]. Consequently, an increasing number of researchers are emphasizing the significance of the immune system, oxidative stress balance, and metabolic alterations in the pathophysiological underpinnings of this disorder [29,33,34,35,36]. For instance, previous research conducted by our team has revealed elevated levels of specific cytokines, including interleukin (IL)-1β, IL-6, tumor necrosis factor α (TNF-α), and transforming growth factor β (TGF-β), in both the splenic and serum samples [29]. This elevation in cytokine levels was found to be associated with reductions in the PFC volume and abnormalities in the dopaminergic system in juvenile SHRs. Ongoing research has yielded varied findings regarding the potential association between ADHD and inflammatory cytokines in pediatric populations [37,38,39]. However, several investigations have documented elevated levels of pro-inflammatory cytokines, notably interleukin (IL)-10, IL-6, IL-1β, and TNF-α, in children diagnosed with ADHD in comparison to their typically developing counterparts [38,39,40,41,42]. Some studies have indicated that cytokines may modulate the activity of neural circuits within the basal ganglia and dopamine (DA) synthesis [43,44]. Furthermore, it is well-established that elevated levels of pro-inflammatory cytokines can significantly influence oxidative stress [45]. Specifically, IL-1β, IL-6, and TNF-α, are recognized for their involvement in promoting oxidative stress through various pathways: inducing Reactive Oxygen Species (ROS) production, disrupting antioxidant defense systems, activating inflammatory signaling pathways, impairing mitochondrial function, and impeding cellular repair mechanisms, contributing to redox imbalance [46,47]. While evidence suggests that ROS may play a role in certain neuropsychiatric and neurodevelopmental disorders, including ADHD, the relationship is complex and not yet fully understood [36,48,49,50,51]. Research has yielded some evidence of redox imbalance in individuals with ADHD, but the findings are inconsistent across all studies. Some studies have reported elevated markers of oxidative stress, such as increased levels of lipid peroxidation and a reduced antioxidant capacity, in individuals with ADHD compared to control groups [36,50]. This state can damage cellular components, including DNA, proteins, and lipids, which may have implications for brain function and structure [52,53]. To date, it is not currently clear whether oxidative stress directly causes ADHD or is a consequence of the disorder.

It is noteworthy to mention some evidence suggests that certain brain regions in individuals with ADHD may exhibit altered metabolism compared to typically developing controls. More precisely, single-photon emission computed tomography (SPECT) or positron emission tomography (PET) has documented decreased metabolic activity in the fronto-striatal regions among individuals diagnosed with ADHD [54,55,56,57]. Moreover, Lou et al. [58,59,60,61] have consistently identified reduced blood flow (hypoperfusion) in the striatal areas using SPECT, especially within the caudate nuclei region in ADHD children. Additionally, the study using fluorodeoxyglucose PET revealed that individuals with ADHD exhibited a decrease in cerebral glucose (G) metabolism in the frontal cortex while they performed an auditory attention task [57]. Similarly, our published findings have revealed elevated G levels and other metabolic markers within the PFC of juvenile SHRs [30]. These findings are in alignment with the research previously mentioned. However, the relationship between these neuroimaging findings and clinical symptoms of ADHD is complex and not fully understood. Neuroimaging studies contribute to the understanding of the neurobiology of this disorder, but the condition remains multifaceted, and its exact mechanisms still needed to be explored.

The aforementioned studies involving individuals with ADHD have enabled the observation of significant anatomical alterations within various brain regions. Nevertheless, exploring ADHD remains challenging for several reasons: heterogeneity due to treatment effects or sample size, especially when investigating age-dependent brain structure abnormalities. Thus, animal models offer a valuable opportunity to see developmental changes in the brain due to the availability of brain tissue, which provides a study of ADHD-related changes across the lifespan in a shorter time frame than humans. Moreover, laboratory settings allow for precise control over environmental variables, such as diet, stress levels, and exposure to toxins, which can help isolate specific factors contributing to ADHD and also eliminate other interfering factors: comorbidities (such as anxiety, depression, and learning disorders), as well as environmental influences (parenting, socioeconomic status, diet, and exposure to toxins). Thus, integrating human and rodent research approaches allows for a more comprehensive understanding of ADHD, its causes, and potential treatments.

To date, the SHR animal model has been extensively validated in terms of descriptive, construct, and predictive validity. The predictive validity of the SHR model in ADHD is supported by evidence that SHRs exhibit hyperactivity, impulsivity, and attention deficits, as well as learning and memory impairments—symptoms of ADHD that are also present in humans [62,63,64]. Additionally, SHRs show age-specific patterns of hyperactivity, impulsivity, and attention deficits, which closely mimic the developmental trajectory of ADHD in humans. For example, symptoms in SHRs tend to emerge during periods corresponding to childhood and adolescence in humans, making them particularly relevant for studying the early onset and progression of this disorder symptoms over time. Additionally, SHRs demonstrate neurobiological correlations with ADHD patients [65]. Specifically, these animals exhibit alterations in dopaminergic signaling, particularly in brain regions such as the motor and prefrontal cortex, which are also involved in the neurobiology of ADHD in humans [66]. SHRs have been found to have reduced dopamine transporter (DAT) density and altered dopamine receptor (DR) function, leading to imbalances in DA levels that are believed to underlie many of the symptoms of ADHD [67,68]. Another piece of key evidence supporting the predictive validity of the studied model, and justifying the use of SHRs in ADHD research, is their ability to respond to drugs used to treat this condition. Specifically, methylphenidate, atomoxetine, and amphetamines have been shown to reduce hyperactivity and improve attention in SHRs, similar to their effects in people with ADHD [68,69].

It is also important to note that this experiment focuses on SHRs aged between 4 to 10 weeks to investigate changes in the striatal regions associated with ADHD. This age range covers key developmental stages in the pathology of this neurodevelopmental disorder. However, it is worth considering whether hypertension (a characteristic feature of SHRs) might influence the results of this study. According to the literature, the early development of hypertension in SHRs begins after 7 weeks of age [70]. In this study, most of the neuroanatomical and biochemical changes were observed between 4 and 7 weeks of age, which allowed us to minimize the impact of hypertension on the results. It is worth noting that adult SHRs also exhibit behavioral and neurotransmitter abnormalities in key brain areas involved in ADHD [71]. However, these alterations differ significantly from those observed in juvenile animals. More specifically, adult SHRs show cognitive dysfunction, increased anxiety, and impaired learning and memory abilities [71]—symptoms that parallel those seen in adults with ADHD [72]. Additionally, adult SHRs display neurotransmitter irregularities that hinder synaptic communication [71], which is consistent with the neurotransmitter imbalances found in adults with ADHD [72]. These abnormalities are particularly evident in brain regions involved in attention regulation, such as the PFC and striatum. Similar to human ADHD, these neurotransmitter disruptions in adult SHRs contribute to persistent difficulties in executive function, emotional regulation, and impulse control. Thus, the adult SHR model mirrors key aspects of ADHD in humans, further strengthening the rationale for using this animal model in ADHD research.

In light of the preceding considerations, the objectives of the current study were to (a) delineate age-related (from 4 until 10 weeks) alterations in the volume of distinct striatal regions (the caudate putamen (CPu), external globus pallidus (EGP), lateral accumbens shell (LaCBsH), and nucleus accumbens core (AcbC)) and the neuronal density within these regions in the SHRs (rat model of ADHD) and WKY rats (control animals) aged 4–10 weeks, which encompass the developmental stages from weaning to the onset of puberty; (b) assess the concentrations of specific immune (interleukin (IL)-1α, IL-1β, IL-6, serine/threonine-protein mammalian target of rapamycin (mTOR), glucocorticoid receptor (GCsRβ), transforming growth factor beta (TGF-β), and RAC-alpha serine/threonine-protein kinase (AKT-1)), oxidative stress (malondialdehyde (MDA), sulfhydryl group (-SH), superoxide dismutase (SOD), peroxidase (POD), glutathione reductase (GSR), and glutathione S transferases (GST)), and metabolic (glucose (G), fructosamine (FrAm), iron (Fe), lactic acid (LA), lactate dehydrogenase (LDH), alanine transaminase (ALT) and aspartate transaminase (AST)) markers in the striatum of juvenile (5 weeks) and maturing (10 weeks) SHRs.

## 2. Results

### 2.1. Volumetric Alternations

#### 2.1.1. Caudate Putamen (CPu)

**Figure 1:**
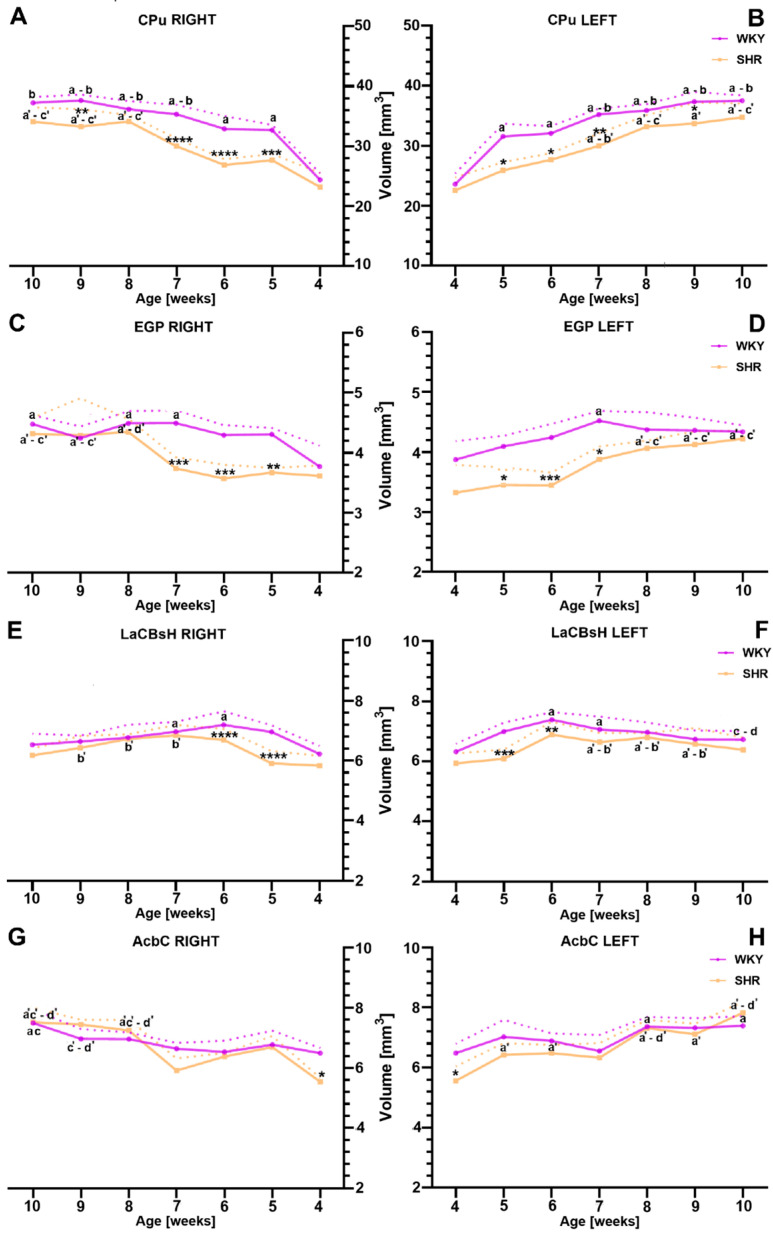
The figure shows the dynamics of changes in the volume of the striatal areas: caudate putamen (CPu; (**A**,**B**)), external globus pallidus (EGP; (**C**,**D**)), lateral accumbens shell (LaCBsH; (**E**,**F**)), and nucleus accumbens core (AcbC; (**G**,**H**)) in the postnatal (4–10 weeks of age) development of SHRs and WKYs (n = 5 or 6 for each strain in each developmental stage studied). Note that the most significant reduction in the volume of striatal regions in SHRs occurs between the 4th and 7th weeks of life. No significant differences in the volumes of this structure were observed between adult individuals of both strains. The differences between strains were indicated by the symbols: *—*p* < 0.05, **—*p* < 0.01, ***—*p* < 0.001, and ****—*p* < 0.0001. Age-dependent developmental differences between animals were denoted as follows: a–d (*p* < 0.05–*p* < 0.001) for the WKY strain, and a’–d’ for SHR strain; and 4 vs. other weeks (a,a’), 5 vs. other weeks (b,b’), and so forth. The dashed lines represent the standard error of the mean (SEM).

The comparative analysis conducted in this study revealed a significantly reduced total volume of the CPu in SHRs at 5, 6, 7, and 9 weeks of age (in both the right and left hemispheres; Figure 1A,B; *p* < 0.05–*p* < 0.0001) compared to age-matched WKYs. However, in both the ADHD animal models and the control group, no statistically significant differences (*p* > 0.05) were observed in the total CPu volume between the right and left hemispheres at the studied age stages (Figure 1A,B). In the SHR group, a significant increase in CPu volume was noted starting from weeks 7/8 (*p* < 0.0001–*p* < 0.01), whereas, in the control group, this increase occurred between weeks 4 and 5 (*p* < 0.05–*p* < 0.0001). Interestingly, in WKYs, the stabilization of CPu volume changes occurred between weeks 6 and 10 (*p* > 0.05).

#### 2.1.2. External Globus Pallidus (EGP)

In parallel with the observations of the CPu, the total volume of the EGP showed a marked reduction in SHRs at 5, 6, and 7 weeks of age, in both the right (Figure 1C; *p* < 0.01–*p* < 0.001) and left (Figure 1D; *p* < 0.05 or *p* < 0.001) hemispheres, compared to age-matched controls. Similarly to the findings in the CPu, no statistically significant volumetric differences (*p* > 0.05) in the EGP were observed between the hemispheres in both SHRs and WKYs (*p* > 0.05). In the SHR group, a more pronounced increase in total EGP volume was noted between 7–8 weeks (right hemisphere) and 6–9 weeks (left hemisphere). Subsequent developmental stages in SHRs did not show significant volumetric changes, with the volume remaining stable. Conversely, in the control group, the most significant increase in total EGP volume occurred between 4 and 5 weeks, after which it remained relatively stable through the 10th week.

#### 2.1.3. Lateral Accumbens Shell (LaCBsH)

Turning to the LaCBsH, a reduced total volume was observed in SHRs at 5 and 6 weeks of age (in both the right and left hemispheres) (Figure 1E,F; *p* < 0.01–*p* < 0.0001) compared to age-matched controls. Consistent with the previously mentioned regions, no significant differences (*p* > 0.05) in LaCBsH volume were observed between the right and left hemispheres in either SHRs or WKYs. In both the SHR and WKY groups, the most significant volume increase occurred by 6 weeks of age, with volumes remaining stable in subsequent stages of life.

#### 2.1.4. Nucleus Accumbens Core (AcbC)

For the AcbC, a reduction in total volume was noticeable only at the 4th week of age in both the right and left hemispheres (Figure 1G,H; *p* < 0.05) in SHRs compared to their control counterparts. Consistent with observations in the CPu, EGP, and LaCBsH, there were no significant volume differences between the hemispheres (Figure 1G,H; *p* > 0.05) in either SHRs or WKYs. Furthermore, throughout the entire lifespan examined (4–10 weeks), the AcbC volumes in both SHRs and WKYs showed no significant differences and remained at similar levels.

### 2.2. Alternations in Neuronal Density Across Distinct Striatal Regions

#### 2.2.1. Caudate Putamen (CPu)

**Figure 2:**
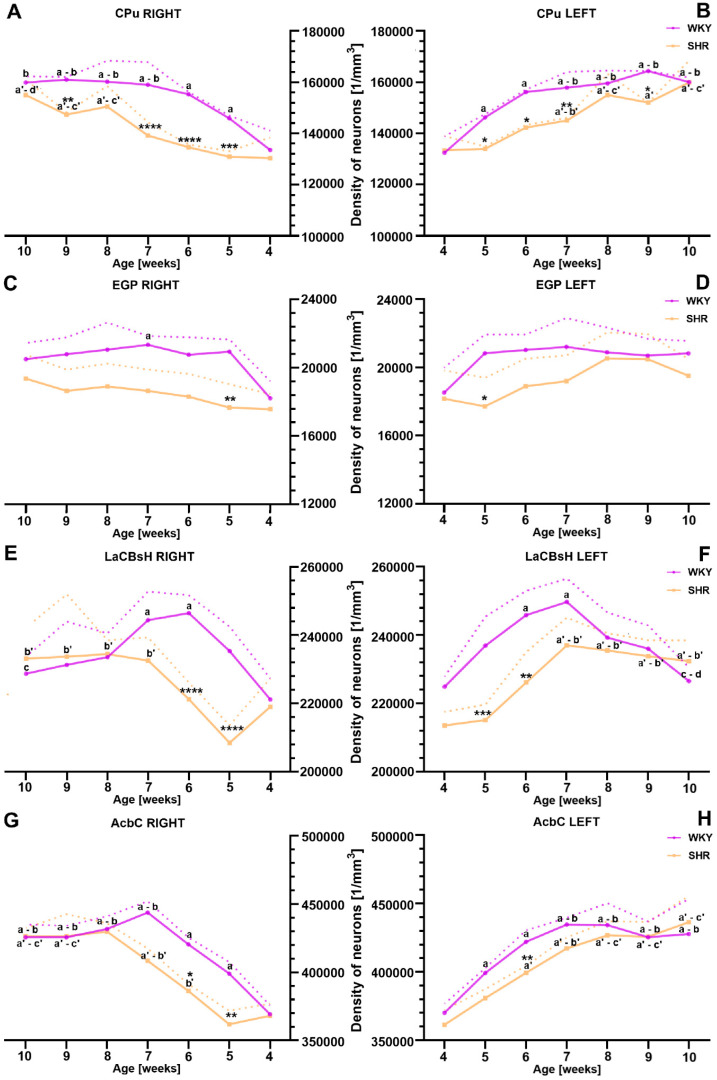
The figure represents a comparison of the mean neuronal density [mm^3^ ] in different areas of the striatum—(**A**) CPu right hemisphere; (**B**) CPu left hemisphere; (**C**) EGP right hemisphere; (**D**) EGP left hemisphere; (**E**) LaCBsH right hemisphere; (**F**) LaCBsH left hemisphere; (**G**) AcbC right hemisphere; and (**H**) AcbC left hemisphere—in the postnatal (4–10 weeks of age) development of SHRs and WKYs (n = 5 or 6 for each strain in each developmental stage studied). It is worth noting that the most pronounced decrease in neuronal density within the striatal regions occurs in juvenile animals (between the 5th and 7th weeks of life). At later stages of development, significant differences were not detected, with the exception of the CPu in the 9th week. The differences between strains were indicated by the symbols: *—*p* < 0.05, **—*p* < 0.01, ***—*p* < 0.001, and ****—*p* < 0.0001. Age-dependent developmental differences between animals were denoted as follows: a–d (*p* < 0.05–*p* < 0.001) for the WKY strain, and a’–d’ for SHRs; and 4 vs. other weeks (a,a’), 5 vs. other weeks (b,b’), and so forth.

The average neuron density in the CPu was significantly lower in SHRs at 5 weeks (right hemisphere *p* < 0.001; left hemisphere *p* < 0.05), 6 weeks (right hemisphere *p* < 0.0001; left hemisphere *p* < 0.05), 7 weeks (right hemisphere *p* < 0.0001; left hemisphere *p* < 0.01), and 9 weeks of age (right hemisphere *p* < 0.01; left hemisphere *p* < 0.05) compared to controls (Figure 2A,B). No significant differences in average neuron density between the hemispheres were observed in either group at any of the studied age stages. Interestingly, SHRs exhibited a characteristic pattern of neuron distribution, with the most significant increase in neuron density occurring between 4 and 8 weeks of age (*p* < 0.01). In later stages of development, the average neuron density stabilized and remained at a similar level (*p* > 0.05). Conversely, in WKYs, the greatest increase in average neuron density was observed between 4 and 6 weeks of age (*p* < 0.05–*p* < 0.01), after which it remained stable until 10 weeks.

#### 2.2.2. External Globus Pallidus (EGP)

Moving to another area of the striatum, the average neuron density in the EGP was significantly reduced in SHRs only at 5 weeks of age, compared to age-matched WKYs (right hemisphere *p* < 0.01; left hemisphere *p* < 0.05; Figure 2C,D). No significant differences in neuron density between the hemispheres were observed in either SHRs or WKYs (*p* > 0.05). Among SHRs, the dynamics of neuron density changes did not differ significantly across the different age stages (*p* > 0.05). Similarly, in WKYs, no significant changes in neuron density dynamics were observed throughout the animals’ lifespan, with densities remaining at similar levels (*p* > 0.05), except in the right hemisphere, where a more pronounced increase in neuron density was noted at 7 weeks of age (*p* < 0.05).

#### 2.2.3. Lateral Accumbens Shell (LaCBsH)

Regarding the LaCBsH, the most significant decrease in neuron density was observed at 5 weeks of age (right hemisphere *p* < 0.0001; left hemisphere *p* < 0.001; Figure 2E,F) and 6 weeks of age (right hemisphere *p* < 0.0001; left hemisphere *p* < 0.01) in SHRs compared to age-matched controls. In SHRs, the most intense increase in neuron density occurred between 5 and 7 weeks of age (*p* < 0.05), with relatively stable densities in the subsequent weeks. In contrast, WKYs exhibited a different pattern of neuron distribution, with the most significant increase in neuron density observed between 4 and 6/7 weeks of age, followed by a noticeable and significant (*p* < 0.05) decrease in density at 9 and 10 weeks of age.

#### 2.2.4. Nucleus Accumbens Core (AcbC)

In the case of the AcbC, a significant reduction in neuronal density was observed in SHRs at weeks 5 (right hemisphere, *p* < 0.01; Figure 2G) and 6 (both right *p* < 0.05 and left hemisphere *p* < 0.01; Figure 2G,H) compared to the age-matched control group. Among the SHRs, the most pronounced increase in neuronal density occurred between weeks 5 and 8 of life. In the subsequent stages of life, the neuronal density in SHRs remained relatively stable. As for the WKYs, the most significant increase in neuronal density was observed between weeks 5 and 7, followed by a similar density thereafter.

### 2.3. Biochemical Alternationss in the Striatum

#### 2.3.1. Inflammatory Markers

**Figure 3:**
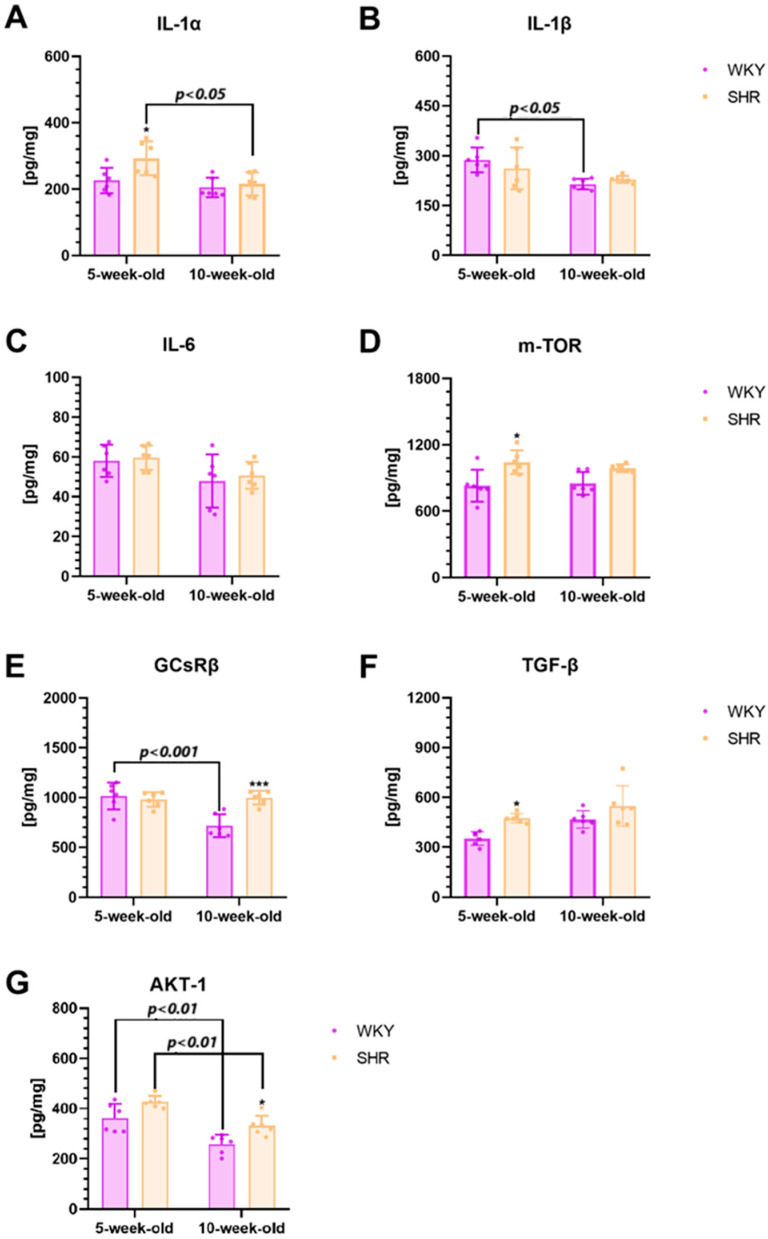
Quantification of interleukin (IL)-1α (**A**), IL-1β (**B**), IL-6 (**C**), serine/threonine-protein mammalian target of rapamycin (mTOR; (**D**)), glucocorticoid receptor (GCsRβ; (**E**)), transforming growth factor beta (TGF-β; (**F**)), and RAC-alpha serine/threonine-protein kinase (AKT-1; (**G**)) levels in striatum supernatants. Notice the increase in immune markers in juvenile SHRs as well as in adult WKYs. Results are presented as the mean ± SEM (n = 6/per group). Significance levels are denoted by * and *** (indicating differences at *p* < 0.05 and *p* < 0.001) between SHRs and WKYs, and *p* < 0.05–*p* < 0.001 indicate distinctions between juvenile and maturing rats within the same strain.

In the current study, significantly elevated levels of IL-1α, m-TOR, and TGF-β were observed in 5-week-old SHRs compared to age-matched controls (Figure 3A,D,F; *p* < 0.05). Notably, GCsRβ and AKT-1 levels were found to be significantly higher in 10-week-old SHRs compared to their age-matched WKYs (Figure 3E,G; *p* < 0.001, *p* < 0.01, respectively). Additionally, IL-1α and AKT-1 levels were significantly higher in 5-week-old SHRs compared to their 10-week-old counterparts (Figure 3A,G; *p* < 0.05, *p* < 0.01, respectively). Conversely, in WKYs, IL-1β, GCsRβ, and AKT-1 concentrations were significantly higher in the 5-week-old animals compared to the 10-week-old ones (Figure 3B,E,G; *p* < 0.05, *p* < 0.001, and *p* < 0.01, respectively).

#### 2.3.2. Oxidative Stress Markers

**Figure 4:**
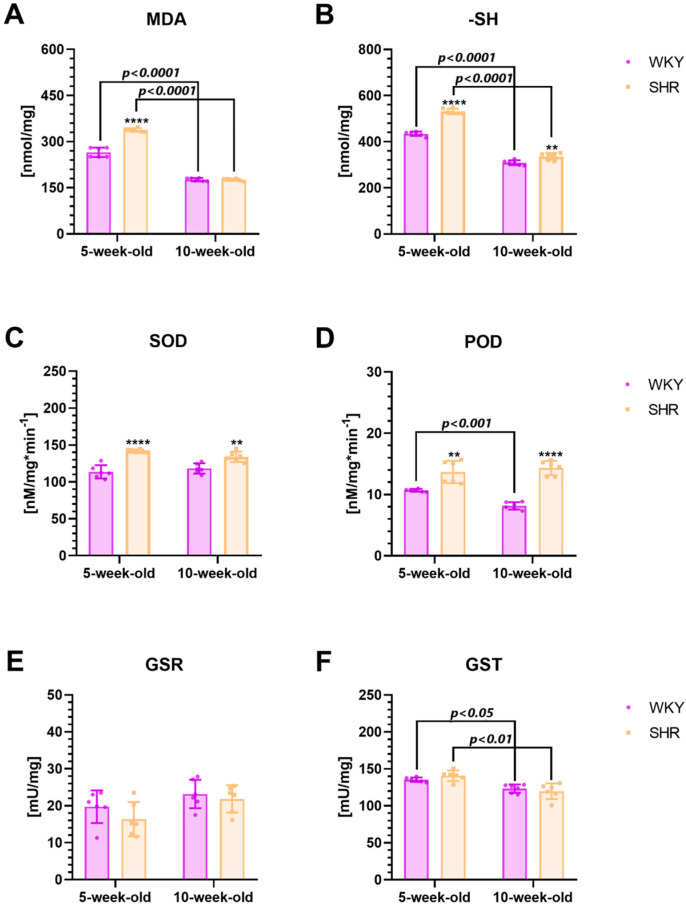
Quantification of malondialdehyde (MDA; (**A**)) and sulfhydryl group (-SH; (**B**)), superoxide dismutase (SOD; (**C**)), peroxidase (POD; (**D**)), glutathione reductase (GSR; (**E**)), and glutathione S transferases (GST; (**F**)) levels/activity in striatum supernatants. Observe the elevation of oxidative stress markers in both juvenile SHRs and adult WKYs. Results are presented as the mean ± SEM (n = 6/per group). Significance levels are denoted by ** and **** (indicating differences at *p* < 0.01, and *p* < 0.0001) between SHRs and WKYs, and *p* < 0.05–*p* < 0.0001 indicate distinctions between juvenile and maturing rats within the same strain.

The concentrations of MDA, -SH, SOD, and POD were significantly elevated in 5-week-old SHRs compared to the age-matched control group, with statistical significance observed (Figure 4A–D; *p* < 0.0001, *p* < 0.0001, *p* < 0.0001, and *p* < 0.001, respectively). Additionally, higher levels of -SH, SOD, and POD were also noted in 10-week-old SHRs compared to their age-matched controls (Figure 4B–D; *p* < 0.01, *p* < 0.01, and *p* < 0.0001, respectively). No significant differences in GST and GSR levels were observed between SHRs and WKYs, regardless of the age studied (Figure 4E,F; *p* > 0.05). Moreover, SHRs exhibited elevated levels of MDA, -SH, and GST at 5 weeks of age compared to the 10-week-old animals (Figure 4A,B,F; *p* < 0.0001, *p* < 0.0001, and *p* < 0.01, respectively). Conversely, WKYs showed significantly higher levels of MDA, -SH, POD, and GST at 5 weeks of age compared to their 10-week-old counterparts (Figure 4A,B,D,F; *p* < 0.0001, *p* < 0.0001, *p* < 0.001, and *p* < 0.05, respectively).

#### 2.3.3. Metabolic Markers

**Figure 5:**
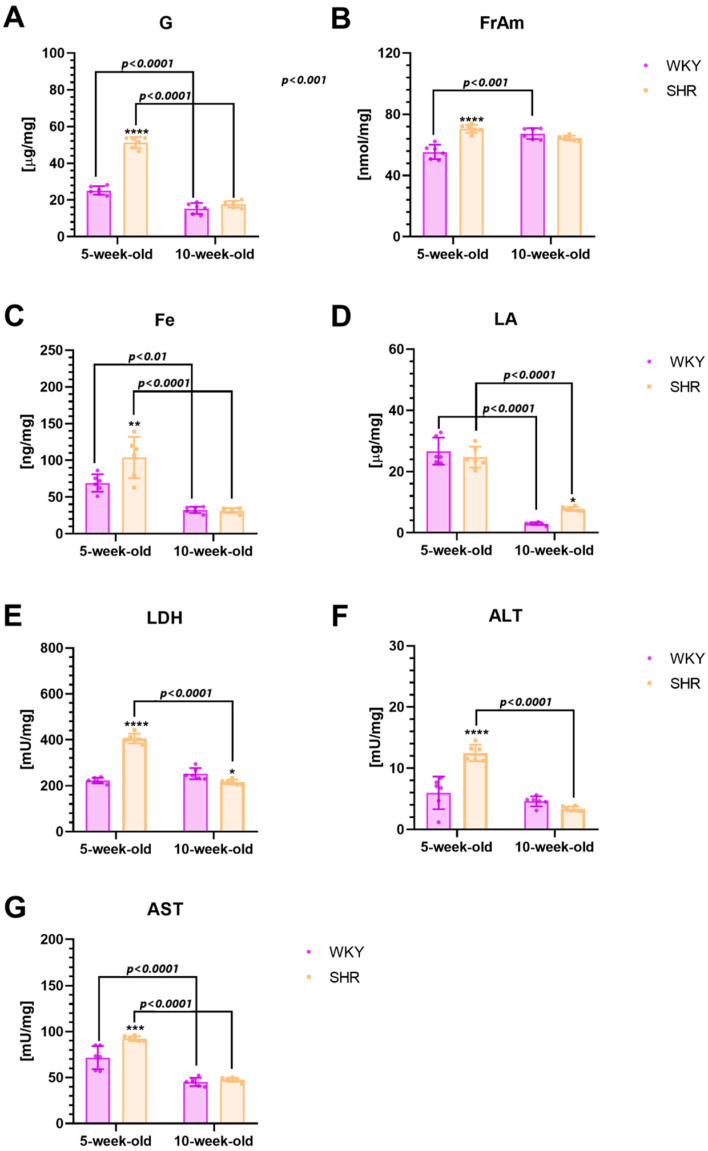
Quantification of glucose (G; (**A**)), fructosamine (FrAm; (**B**)), iron (Fe; (**C**)), lactic acid (LA; (**D**)), lactate dehydrogenase (LDH; (**E**)), alanine transaminase (ALT; (**F**)), and aspartate transaminase (ALT; (**G**)) levels/activity in striatum supernatants. Take a look at the increase in metabolic markers in both young SHRs and adult WKYs. Results are presented as the mean ± SEM (n = 6/per group). Significance levels are denoted by *, **, ***, and **** (indicating differences at *p* < 0.05, *p* < 0.01, *p* < 0.001, and *p* < 0.0001) between SHRs and WKYs, and *p* < 0.01, *p* < 0.001, and *p* < 0.0001 indicate distinctions between juvenile and maturing rats within the same strain.

Our study revealed significantly elevated levels/activities of G, FrAm, Fe, LDH, ALT, and AST in 5-week-old SHRs compared to age-matched WKYs (Figure 5A–C, E–G; *p* < 0.0001, *p* < 0.0001, *p* < 0.01, *p* < 0.0001, *p* < 0.0001, and *p* < 0.001, respectively). Additionally, the LDH level was significantly lower in 10-week-old SHRs compared to age-matched controls (Figure 5E; *p* < 0.05). In turn, the LA level was higher in mature SHRs in comparison to age-matched WKYs (Figure 5D; *p* < 0.05). Notable developmental differences were also observed. Specifically, SHRs exhibited significantly higher levels/activities of G, FrAm, Fe, LA, LDH, ALT, and AST at 5 weeks of age compared to 10 weeks (Figure 5A, C–G; *p* < 0.0001, respectively). Conversely, WKYs showed higher levels/activities of G, Fe, LA, and AST at 5 weeks compared to 10 weeks (Figure 5A,C,D,G; *p* < 0.0001, *p* < 0.01, *p* < 0.0001, and *p* < 0.0001, respectively). It is worth noting that in 5-week-old WKYs FrAm levels were significantly lower than in 10-week-old individuals of the same strain (Figure 5B; *p* < 0.001).

## 3. Discussion

### 3.1. Striatal Volume and Neuron Density Alternations

The presented results provide, for the first time, detailed evidence of a significant volume reduction in the striatum areas—caudate putamen (CPu), external globus pallidus (EGP), accumbens shell (LaCBsH), and nucleus accumbens core (AcbC)—in juvenile and maturing spontaneously hypertensive rats (SHRs), an animal model of ADHD. While comprehensive volumetric measurements of specific striatal areas in SHRs have not been conducted so far, the finding by Hsu et al. [31] showed corresponding results, where a notable decrease in the total striatal volume was observed in young SHRs [31]. Additionally, similar patterns have been identified in neuroimaging studies of ADHD patients, indicating a reduction in the volume of the caudate, putamen, and globus pallidus during childhood and early adolescence [24,73,74,75]. Furthermore, the observed developmental trajectory of volume alterations of the striatal areas in SHRs appears to be very similar to those observed in magnetic resonance imaging (MRI) studies among ADHD patients [10,75,76,77]. The observed developmental changes may provide a potential explanation for previous findings, suggesting that alterations in striatal areas’ volume in ADHD normalize with age. Similarly, we observed no significant differences in the volumes of the striatal areas in maturing SHRs, which is supported by previous findings considering the same animal model [30,31]. Additionally, it remains consistent with previous studies among adult ADHD patients, which presented no differences in basal ganglia volumes [78,79,80]. However, there is also divergent evidence suggesting both larger [75] and smaller [80,81] volumes of striatal areas in adult ADHD patients compared to healthy controls. This variability may arise from differences in sample characteristics, such as age, sex, and ADHD subtype, as well as methodological differences in imaging techniques and analysis approaches [82]. Notably, the reduction in neuronal density observed in 9-week-old SHRs might indicate an enlargement of the brain ventricles, as reported in other studies involving SHRs of the same age [30,83]. Moreover, we did not observe significant differences in the volumes of the striatal areas between the hemispheres, which is consistent with previous studies [84]. Nevertheless, there is evidence to support right-side reductions in the putamen, globus pallidus, and caudate [85,86], and also left-side caudate [12,87,88] reductions in ADHD. The variations in findings regarding volume alternations in adults and hemispheric differences in ADHD could stem from several factors, including the diversity of research methodologies, the sample characteristics, and the complexity of this disorder as a neurodevelopmental disorder.

In addition to the volumetric alternations noted in the striatal areas in the ADHD rat model, our developmental study revealed a distinct shift in neurons’ distributions within the striatal areas of SHRs when compared to the control group throughout the studied animals’ lifespan. Specifically, we observed a reduction in neuronal density in most striatal areas, namely, CPu, EGP, LaCBsH, and AcbC, among juvenile SHRs, when compared to their control counterparts. Comparing our results with the existing literature is particularly challenging, considering that our study is the first to analyze neuron densities in the striatum regions using an animal model of ADHD. Nonetheless, we will strive to contextualize our results within the existing body of literature. Firstly, our results align with the current evidence and our previous study, indicating a reduction in grey matter density in other brain regions associated with motor control, i.e., the PFC and motor cortex in juvenile SHRs [29,30]. Furthermore, an important support for our findings are studies using Proton Magnetic Resonance Spectroscopy, which enables the reliable non-invasive detection of intracerebral neurochemical concentrations in vivo [89]. N-acetylaspartate (NAA) stands as a pivotal metabolite, reflecting the quantity and functional status of neurons in the targeted brain region [90,91]. A low level of NAA is considered an indicator of neuronal loss or significant neuronal dysfunction [92]. Namely, the finding by Jin et al. [93] provides data that the mean NAA peak in children with ADHD was significantly lower than in healthy controls in the bilateral striatum [93,94] and PFC [95]. In contrast, in adult ADHD patients, NAA peaks did not differ between ADHD patients and controls [96], which supports our observations. Additional support for our results is provided by reports using the Diffusion Tensor Imaging (DTI) method which is a valuable tool for characterizing the microstructural tissue integrity and fiber pathways based on the water diffusion properties; it primarily provides insights into the structural aspects of white matter, such as axonal integrity and myelination [97]. A reduction in fractional anisotropy (FA) is often interpreted as a disruption or alteration in the microstructural integrity of white matter tracts. This could be due to various factors, including demyelination, axonal damage, or changes in tissue density. In neurological and psychiatric conditions, reduced FA is frequently observed and may signify abnormalities in the underlying neural architecture, which can contribute to functional and cognitive changes [98]. Namely, a study provided by Ashtari et al. [99] showed that children with ADHD had decreased FA in, among others, the premotor, striatal areas [99]. Similarly, de Zeeuw et al. presented reduced microstructural organization in the fronto-striatal regions in ADHD children [100].

Finally, it is worth noting that the presented alterations in the volume and microstructure of the striatum may be the cause of the impaired structural connectivity in the fronto-striatal network modulating performance in tasks involving cognitive control [101,102], an ability typically impaired in ADHD [103], and also the hypoactivity of this structure observed in functional magnetic resonance imaging (fMRI) studies in ADHD children when compared to healthy controls [104,105,106]. The structural and functional changes observed in the striatal areas of juvenile ADHD individuals may significantly impact the symptoms of the disorder, as also demonstrated in SHRs. Specifically, previous studies of locomotor activity in SHRs and WKYs showed that parameters such as the horizontal activity, total distance, and movement time were significantly the highest in SHRs compared to WKYs at 5–7 weeks of age [107,108]. Similarly, this period also aligns with the peak intensity of ADHD symptoms observed in children with ADHD [28,109]. Moreover, dysregulated DA signaling in the striatum is linked to impaired reward processing and heightened impulsivity, contributing to difficulties in delaying gratification and making choices considering long-term consequences in individuals with ADHD [110,111].

Interestingly, the peak period of significant structural abnormalities in the striatum of SHR rats is observed during the prepubescent phase, particularly between the fourth and seventh weeks of life. This coincides with the process of synaptic pruning, suggesting that plasticity mechanisms may be responsible for observed developmental changes associated with ADHD [112,113]. This timeframe (equivalent to 6–10 years of age in humans [114]) correlates with the most noticeable changes in the striatum and caudate nucleus, which are smaller and show reduced activity in children with ADHD compared to healthy peers [10,77,115,116]. These changes may underlie difficulties with attention and self-control—key symptoms of this disorder observed in both SHR rats [108,117] and ADHD children [118].

It is worth adding that the decrease in neuronal density in juvenile SHRs (4–7 weeks), followed by an increase in density in maturing individuals (8–10 weeks) of this strain, may suggest the presence of compensatory mechanisms in the striatum of SHRs. This could be the result of increased antioxidant levels and metabolic alterations occurring in the striatum.

### 3.2. Inflammatory Markers

The results presented in this study highlight a significant increase in the levels of IL-1α, m-TOR, and TGF-β in the striatum of juvenile SHRs compared to age-matched WKYs. Additionally, the levels of GCsRβ and AKT-1 were significantly higher in maturing SHRs than in their WKY counterparts. Understanding the immune environment of the striatum in ADHD is particularly important due to the role cytokines play in various neurological disorders. While comprehensive data on immune markers in the SHRs’ striatum are lacking, some studies support our observations. Elevated levels of pro-inflammatory cytokines such as IL-1α, IL-1β, IL-6, TNF-α, m-TOR, and MCP-1 have been previously reported in juvenile SHRs in other brain regions involved in ADHD, including the prefrontal cortex (PFC) and hippocampus [30,119]. Moreover, Donfrancesco et al. reported increased levels of IL-6 and IL-10 in the serum of children with ADHD [38].

Interestingly, we did not observe significant differences in IL-1β levels in the striatum between the tested strains, regardless of age. Similarly, a study by Leffa et al. found no differences in IL-1β levels between SHRs and WKYs of the same age, and also our previous research showed no significant alterations in IL-1β levels in the PFC of both young and maturing SHRs [30,120]. Nevertheless, presented findings suggest ongoing inflammation in the striatum, which could contribute to the observed morphometric changes, and functional abnormalities in this region contributing to ADHD-specific symptoms.

Specifically, pro-inflammatory cytokines such as IL-1α activate signaling pathways like Nuclear Factor kappa B (NF-κB) and Mitogen-Activated Protein Kinase (MAPK) [121]. Chronic NF-κB activation in neurons or neighboring glial cells promotes neurotoxic effects by increasing the production of pro-inflammatory cytokines [122]. IL-6 overproduction amplifies the inflammatory environment, leading to prolonged microglial and astrocytic activation, which damages neurons by inducing oxidative stress, promoting the production of reactive oxygen species (ROS), and damaging neuronal structures such as membranes, proteins, and DNA. The inflammatory cytokines can also impact neuronal survival [122]. Additionally, NF-κB activation may exacerbate excitotoxicity, a process where the excessive stimulation of neurons by excitatory neurotransmitters (mainly glutamate) leads to neuronal death. Pro-inflammatory cytokines such as IL-1α and IL-6 also sensitize glutamate receptors, especially NMDA receptors, resulting in an excessive calcium influx into neurons [122,123]. Elevated intracellular calcium activates enzymes that degrade cellular components, leading to apoptosis or necrosis. Moreover, an excessive calcium influx and excitotoxicity impair mitochondrial function, resulting in metabolic disturbances, reduced ATP production, increased ROS generation, and the activation of cell death pathways [123]. Similarly, the chronic activation of MAPK signaling pathways, such as p38 MAPK, JNK, and ERK, can contribute to neuronal damage by promoting pro-apoptotic pathways and inducing the expression of inflammatory genes [124]. Notably, JNK and p38 MAPK are highly sensitive to oxidative stress, a major byproduct of brain inflammation. These kinases induce mitochondrial dysfunction by promoting ROS production and activating mitochondrial pro-apoptotic pathways, leading to the release of apoptosis-inducing factors [124].

Furthermore, elevated levels of cytokines such as TNF-α, IL-1α, IL-1β, and IL-6 have been shown to reduce the synthesis, secretion, and availability of DA and alter the DA receptor expression or function in the brain [125,126]. Reduced DA levels in the striatum have been reported in both juvenile SHRs [63,127,128], and children with ADHD [110,129], and are associated with impaired attention regulation and motor control. Interestingly, the administration of anti-inflammatory agents has been shown to reduce ADHD symptoms in both juvenile SHRs and ADHD patients [130].

The elevated levels of m-TOR and TGF-β observed in the striatum of juvenile ADHD subjects in this study may have significant implications for neuronal function, survival, and neurotransmission. Both molecules are involved in key cellular processes, and their dysregulation could contribute to neurological dysfunction in ADHD. The m-TOR pathway, a critical regulator of cell growth, metabolism, protein synthesis, and synaptic plasticity, is particularly notable [131,132]. Elevated m-TOR activity in the striatum may disrupt neuronal function and signal transmission, leading to excessive or aberrant synaptic remodeling that disturbs the balance of excitatory and inhibitory neurotransmission, particularly impacting DA signaling [131,132,133]. Chronic m-TOR hyperactivation is also associated with neuronal stress and impaired cell health, potentially compromising long-term neuron viability and contributing to cognitive and behavioral deficits characteristic of ADHD [134].

Similarly, TGF-β, a cytokine involved in cell growth regulation, immune response, and brain function, plays a critical role in synaptic plasticity, neuroinflammation, and neuronal survival [135]. Elevated this cytokine levels in the striatum may indicate neuroinflammatory processes that could harm neuronal health and DA signaling [136]. Dysregulated TGF-β signaling can interfere with synapse development and plasticity, altering neurotransmitter release and reuptake, thus exacerbating ADHD symptoms [135,136,137]. Furthermore, TGF-β has dual roles in neuronal survival, where its elevated levels might promote neuroprotection or, when overexpressed, contribute to neurodegeneration [135,137]. TGF-β is also involved in activating astrocytes and microglia, and their overactivation could impair synaptic transmission and neuronal health, further complicating the functional dynamics of the striatum in ADHD [137].

Interestingly, we also observed elevated levels of GCsRβ and AKT-1 in maturing SHRs compared to age-matched WKYs. Supporting our data, a study by van der Meer et al. showed the overexpression of the NR3C1 9β gene encoding GCsRβ in adolescents and young adults with ADHD [138]. Notably, the elevated level of AKT-1 and GCsRβ in maturing SHRs was accompanied by an increase in striatal volume and neuronal density in these areas. A detailed literature review reveals that the AKT-1 signaling pathway is crucial for promoting neuronal survival by inhibiting apoptosis and activating survival pathways [139]. This is achieved through the phosphorylation and inactivation of pro-apoptotic proteins and the activation of anti-apoptotic factors. Under conditions of stress or damage, AKT-1 activation helps neurons resist injury and supports their survival, playing a key role in both neuronal survival and synaptic plasticity [139]. The elevated levels of AKT-1 and GCsRβ in maturing SHRs also suggest increased metabolic activity in the striatum [140,141]. These molecules are involved in various metabolic processes, including insulin signaling, glycolysis, and oxidative phosphorylation [140,141,142]. AKT-1, in particular, plays a key role in regulating glucose uptake and metabolism in cells [142]. Similarly, GCsRβ affects energy metabolism by mediating the actions of glucocorticoids [141]. Interestingly, our results indicate that increased levels of AKT-1 and GCsRβ in maturing SHRs are associated with reduced glucose levels.

It is worth emphasizing that no significant differences were observed in other inflammatory markers, such as IL-1α, IL-1β, IL-6, mTOR, and TGF-β, in adult SHRs, which is consistent with previous studies [120]. This may be due to the elevated levels of cortisol and corticosterone observed in the serum and adrenal glands of adult SHRs [29,35]. As is well known, glucocorticoids are adaptive hormones that exhibit immunosuppressive effects by lowering the levels of pro-inflammatory cytokines [143].

### 3.3. Oxidative Stress Markers

Oxidative stress is a condition that arises when the body’s natural defense mechanisms against harmful molecules called free radicals are overwhelmed by an excessive production of these radicals. ROS constitute the primary type of free radicals, with a significant portion generated within the mitochondria during the process of oxygen metabolism [144]. The current body of research suggests a connection between the pathophysiology of ADHD and an imbalance in oxidative stress. Nonetheless, the precise mechanisms underlying this association remain elusive. It should be noted that our present results reveal significantly increased levels of MDA, a biomarker of oxidative stress, a product of lipid peroxidation [145], in the striatum of juvenile SHRs compared with control peers. In some studies, elevated levels of this dialdehyde have been observed in individuals with ADHD. However, the results regarding the relationship between MDA levels and ADHD are still inconclusive. For example, elevated levels of MDA as well as reactive oxygen species have previously been reported in both the PFC and serum of juvenile SHRs [30,69], and this remains consistent with our findings. At the same time, there is evidence of elevated MDA levels in the serum of ADHD children [146,147]. The opposite data have been presented by Oztop et al. [50] where lower levels of MDA have been reported in children with this disorder [50]. The discrepancies in the literature data on MDA levels in children with ADHD could be attributed to variations in sample demographics, methodologies employed for MDA measurement, and the presence of confounding factors such as diet, comorbidities, or medication usage. Additionally, differences in study designs, including sample size, control group selection, and statistical analyses, may contribute to the observed inconsistencies in the reported MDA levels. Nevertheless, we suggest that elevated MDA levels in juvenile SHRs may contribute to the severity of ADHD-like symptoms in this strain [107], which is supported by the existing literature [145]. Namely, oxidative stress can disrupt the balance of neurotransmitters, especially DA, which is most sensitive to oxidative stress [148]. DA plays a crucial role in regulating attention, impulsivity, and hyperactivity—core symptoms of ADHD. Heightened oxidative stress may impair DA synthesis, release, reuptake, and metabolism, leading to the dysregulation of this catecholamine levels in key brain regions implicated in ADHD, such as the PFC and striatum. This contributes to the severity of ADHD symptoms, which has been shown in prior studies [149].

In response to elevated oxidative stress levels, the organism typically upregulates the synthesis and expression of endogenous antioxidants. Antioxidants are biochemical compounds that act to mitigate the deleterious effects of ROS, which are generated as byproducts of oxidative stress [150]. Similarly, in our study, the increase in MDA levels in juvenile SHRs was accompanied by a significant increase in the -SH group, as well as SOD and POD activity in these animals. These -SH groups are capable of donating electrons to ROS, neutralizing them, and preventing oxidative damage to cells and tissues. The literature data on the level of -SH groups in individuals with ADHD are inconclusive. Specifically, our previous studies have shown elevated levels of -SH groups in juvenile SHRs in the PFC and spleen [29,30]. Similarly, there is evidence indicating higher levels of these groups in the saliva and serum of children with ADHD [50,151]. On the other hand, Öğütlü et al. [152] presented contrasting findings, indicating decreased serum -SH levels in children with ADHD compared to non-ADHD individuals, thus introducing conflicting data into the current body of evidence [152]. The existing literature underscores the crucial role of dynamic thiol/disulfide homeostasis in antioxidant mechanisms, apoptosis, detoxification, cellular signal transduction, and the regulation of enzyme and transcription factor activity [153,154,155]. When it comes to SOD and POD, they are widely recognized as key antioxidant enzymes that play a crucial role in maintaining oxidative balance [156], which is disrupted in individuals with ADHD [36]. Unfortunately, elucidating the implications of elevated SOD and POD activity in juvenile SHRs in the striatum proves challenging due to a lack of available data in existing studies on this matter. Nonetheless, our previous study presented elevated levels of these enzymes in the PFC of juvenile SHRs compared to the WKY counterparts. Moreover, the existing literature supports a significant increase in the total antioxidant status (TAS) in children and adolescents with ADHD [157]. TAS is a measure of the overall antioxidant capacity of a biological sample, such as blood serum, which confirms our findings [157].

Moving on to the other oxidative stress markers investigated in our study, we did not observe any significant differences in the activity of GSR and GST, in the striatum between SHRs and WKYs, regardless of the age group studied. Similarly, the finding by Leff et al. showed no differences in glutathione peroxidase (GPx) levels in the striatum of adult SHRs [120]. These enzymes, i.e., GSR, GST, and GPx, are components of the cellular antioxidant defense system. They work together to maintain a redox balance and protect cells from oxidative damage.

Interestingly, there are also conflicting literature data. For example, studies conducted among ADHD children show no significant differences in the antioxidant levels in the blood of children with ADHD [50,158,159]. Similarly, a meta-analysis indicated that the association between ADHD and elevated antioxidant status was not significant [34]. These discrepancies may be attributed to the different types of tissue analyzed and the species-specific differences in the studied populations [160].

It is important to highlight that our results suggest a decline in the levels of MDA as SHRs age, eventually reaching values statistically comparable to those of WKYs at 10 weeks old, aligning with earlier findings [29,30,120]. Moreover, we observed a sustained significant increase in the levels of antioxidants such as -SH groups, SOD, and POD in adult SHRs compared to age-matched controls. This phenomenon might be a result of compensatory adaptations [161].

Oxidative stress in the striatum, most likely resulting from inflammatory processes, can affect neuron morphology, migration, and plasticity, further influencing the manifestation of ADHD symptoms [36,162]. This is in line with our data, which indicate that heightened oxidative stress coincide with a decrease in the volume of striatal structures, along with a reduction in neuronal density within this region. It is important to emphasize that elevated oxidative stress can affect neuronal metabolism and exacerbate ADHD symptoms [163]. Confirming our thesis, existing reports indicate that treatment with anti-inflammatory and antioxidant agents reduces inflammatory parameters and oxidative stress in both SHRs [119], and ADHD patients [164,165,166], and also leads to a significant reduction in ADHD symptoms [119,164,165,166].

### 3.4. Metabolism Markers

Oxidative stress has the potential to harm cellular structures, encompassing cell membranes and mitochondria, while also diminishing insulin sensitivity. This can impede the cells’ ability to uptake glucose (G) from the bloodstream, ultimately contributing to heightened G levels in tissues [167,168]. Similarly, in the presented study, significantly elevated oxidative stress markers were associated with increased G levels in juvenile SHRs compared to age-matched controls. Our results are consistent with those reported by Ernst et al. [169], who observed a decrease in global metabolism G within the brains of children with ADHD using PET analysis [169]. The reduction in G metabolism during brain development may stem from mitochondrial dysfunction, which is commonly reported in children with ADHD and autism spectrum disorder [170,171]. In the current study, we also observed elevated levels of fructosamine (FrAm) in juvenile SHRs, which is consistent with a previous report showing elevated FrAm levels in the PFC of SHRs at the same age [30]. FrAm is a compound formed as a result of the non-enzymatic glycation of proteins by G. Elevated FrAm levels indicate persistent hyperglycemia over the past 2–3 weeks [172]. However, elevated G levels in the brain may influence the dysregulation of neurotransmitter systems such as dopaminergic pathways [173] and also lead to alterations in brain structure and function, which may contribute to the exacerbation of ADHD symptoms [174]. Moreover, observed in the current study, elevated G levels in the striatum can lead to an increased activity of lactate dehydrogenase (LDH). The primary process of G metabolism in the brain is glycolysis, during which G is converted to pyruvate [175]. In the case of excess pyruvate, which can occur with elevated G levels, cells, through the activity of LDH, can convert excess pyruvate to lactate (LA). This process, known as lactate fermentation, can occur under conditions of hypoxia or when the electron flow in the respiratory chain is limited [175]. Similarly, in the current study, we observed significantly increased striatal LDH activity observed in juvenile SHRs compared to controls. Increased LDH activity leads to elevated LA production, resulting in an increased demand for monocarboxylate transporter 1 (MCT1) to remove excess lactate from cells and maintain homeostasis. This is consistent with previous studies by Medin et al. [176], demonstrating the increased expression of the LA transporter MCT1 in brain microvessels in juvenile SHRs [176]. Interestingly, in our study, a significant increase in LA levels was observed only in maturing SHRs. This phenomenon may be attributed to observed elevated levels of AKT-1, which is associated with LA synthesis in astrocytes [177]. It is worth mentioning that LA plays a diverse role in brain energetics. Firstly, it supports energy production in nerve cells, leading to synaptic remodeling, increased axonal excitability, and the facilitation of memory formation [178]. These actions may explain the presence of compensatory mechanisms that counteract anatomical and physiological abnormalities observed in the ADHD brain. In some conditions, elevated levels of LA may contribute to the formation of lactic acid–calcium complexes, which can downregulate synaptic transmission in the GABAergic system and neuronal function, what have been found in children [179] and also in adult ADHD patients [180,181,182]. The exact role of LDH and LA in the brain function of people with ADHD is still under active debate and requires further research. Nevertheless, increased LA production and increased LDH activity may correlate with impaired homeostasis and the escalation of oxidative stress in the brain [183,184]. Elevated concentrations of these substances may cause excessive ROS production, which may result in cell and mitochondria damage [184].

Moreover, we observed heightened activities of alanine transaminase (ALT) and aspartate transaminase (AST) in juvenile SHRs when compared to age-matched controls. Similarly, elevated levels of these enzymes were previously observed in the serum and PFC of juvenile SHRs [30,185]. These factors serve as common indicators of necrosis, cell damage, and mitochondrial dysfunction. The elevation in ALT and AST activity could be attributed to oxidative stress, known to inflict damage on both cells and mitochondria [186,187]. Reactive oxygen species generated during oxidative stress can disrupt mitochondrial membranes and enzymes, potentially leading to the release of these enzymes into the bloodstream. Furthermore, Skalny et al. [188]. reported increased levels of the amino acids alanine and asparagine in the serum of children with ADHD [188], a phenomenon possibly linked to heightened ALT and AST activity.

Additionally, our study revealed elevated levels of iron (Fe) in the striatum of juvenile SHRs compared to age-matched controls. Fe plays a pivotal role in cellular function and maintaining metabolic balance [189]. Primarily, it is essential for the optimal functioning of the mitochondrial respiratory chain, the primary source of cellular energy. Furthermore, it facilitates electron transfer within this chain, leading to energy production, and plays a crucial role in protecting cells from oxidative stress by participating in the activity of antioxidant enzymes such as catalase and glutathione peroxidase [189,190]. It is significant to note that prolonged iron overload in the striatum of young SHRs could potentially instigate neurodegenerative changes within this brain region. These alterations may include oxidative stress, lipid peroxidation, blood–brain barrier disruption, and neuronal death, as has been previously observed in Alzheimer’s disease patients [191,192,193,194]. Therefore, the increased Fe levels in the striatum of maturing SHRs may result from the heightened oxidative stress and structural damage observed in the striatal areas in our study [195]. This finding is consistent with previous research indicating elevated Fe and ferritin levels following hypertensive or ischemic brain injury in SHRs [196,197]. It should be mentioned that the available literature data on Fe levels in patients with ADHD present conflicting findings. For instance, Chen et al. [198] observed significantly elevated Fe levels in children with ADHD, while other studies indicate no differences in Fe levels in children and adults with this disorder [199,200]. Discrepancies in Fe levels in human studies on ADHD may arise from the fact that Fe levels in pathologically altered tissues could be higher than in peripheral tissues such as blood, plasma, and serum. Additionally, variations in methodological approaches to Fe measurement and the presence of comorbidities or medication use could also contribute to the observed differences. Furthermore, dietary factors, exposure to environmental toxins, and genetic influences on Fe metabolism may play a role in these differences [201,202]. Therefore, careful consideration and control of these factors are crucial for interpreting and comparing results across studies.

Additionally, it should be emphasized that the observed elevation of most metabolic markers in juvenile SHRs compared to age-matched control animals may result from the fact that the SHR strain exhibits increased sympathetic activity compared to WKYs, which could influence metabolic differences.

Interestingly, we observed elevated levels of LA combined with reduced LDH activity in 10-week-old SHRs, which may suggest a disruption in glycolytic metabolism and energy homeostasis. Reduced LDH activity could impair the conversion of LA into pyruvate, leading to LA accumulation and potential mitochondrial dysfunction, which is associated with oxidative stress and neuroinflammation [203].

## 4. Materials and Methods

### 4.1. Animals

The selection of animal strains was conducted with meticulous care to ensure the highest reliability in the results. Additionally, it is worth noting that the identical rat strains were systematically evaluated in our previous research [29,30,31,35,66,107,204]. Importantly, no statistically significant distinctions in mean body weight were detected between the test group (SHRs) and the control group Wistar Kyoto rats (WKYs) at corresponding age stages (Table 1). Importantly, an identical number of sections per animal was consistently analyzed, contingent upon the striatal region under study and the developmental stage. The starting and ending points for each assessed brain remained constant throughout.

#### 4.1.1. Animals with Transcardial Perfusion

Two cohorts of male rats (WKYs as a control group and SHRs as an animal model for ADHD) aged 4–10 weeks (seven distinct age stages; n = 5–6, for each strain at each age) were used to trace age-series alterations in brain morphometry. All rats involved in this study (aged 3 weeks) were acquired from Charles River Laboratory Germany GmbH (Sulzfeld, Germany). Subsequently, the animals were conveyed to the animal house at the Institute of Animal Reproduction and Food Research of the Polish Academy of Sciences in Olsztyn (Olsztyn, Poland), where they were accommodated in threes (to avoid potential isolation-related stress) in accordance with standard laboratory conditions: light/dark cycle (12/12 h), ventilation (12–20 exchanges/h), and temperature (21 ± 1 °C). All rats were provided ad libitum access to drinking water and the same grain mixture (VRF1 diet, Charles River Laboratories, Germany). All animal procedures were approved by the Local Ethics Committee for Animal Experimentation at the University of Warmia and Mazury in Olsztyn, Poland (permission number: no. 43/2014). Animal care and handling strictly adhered to the European Union Directive for animal experiments (2010/63/EU). Every effort was made to minimize animal suffering and limit the use of animals to the minimum necessary to obtain accurate scientific data.

#### 4.1.2. Animals Without Transcardial Perfusion

Twenty-four rats, aged 3 weeks, were procured from Charles River Laboratory Germany GmbH (Sulzfeld, Germany), and subsequently transferred to the animal house at the Institute of Animal Reproduction and Food Research of the Polish Academy of Sciences in Olsztyn, Poland. All animals were consistently provided the same housing, nutrition, and hydration conditions as detailed earlier. Within each group (WKYs and SHRs), animals were analyzed at two distinct developmental stages (n = 6 at each age stage), 5 weeks (juvenile) and 10 weeks (maturing), to investigate differences in immunological, oxidative stress, and metabolic marker content within the striatum. Animal care and handling were conducted in strict conformity with the regulations stipulated by the European Union Directive pertaining to animal experimentation and the 3Rs principles, which stand for Replacement, Reduction, and Refinement. The experiments were meticulously reported in adherence to the ARRIVE guidelines, underscoring our commitment to minimizing any potential suffering or distress experienced by the animals.

### 4.2. Preparation of Brains

#### 4.2.1. Preparation of Brains for Neuron-Specific Nuclear Protein (NeuN) Staining

After undergoing a standard habituation phase (7 days), both SHRs and WKYs were subjected to identical experimental protocols. The rats were deeply anesthetized via intraperitoneal administration of pentobarbital (Morbital, Biowet, Puławy, Poland; 50 mg/kg body weight) in accordance with the guidelines provided by the Humane Society Veterinary Medical Association. After the cessation of respiration, they underwent transcardial perfusion with 0.9% saline, followed by a solution of 4% paraformaldehyde (PFA; pH 7.4; 1040051000, Merck, Darmstadt, Germany) in phosphate-buffered saline (PBS; P5493, Sigma-Aldrich, Darmstadt, Germany) to maintain the natural state of the tissue. After all, the brains were dissected from the skulls and subjected to post-fixation in 4% PFA for 24 h. Following this, the brains were thoroughly rinsed three times with 0.1 M PBS (pH = 7.4, 4 °C), and then cryoprotected (4 days) in a series of graded sucrose (363-117720907, ALCHEM, Toruń, Poland) solutions (10%, 20%, and 30%) at 4 °C. After all, the coronal sections (10 μm thickness each), were precision-cut using a cryostat. These brain sections were then kept attached to the object slides and safely stored at −80 °C for subsequent tissue processing.

#### 4.2.2. Neuron-Specific Nuclear Protein (NeuN) Staining

Following the fixation process, the brains were sliced into 10 µm-thick sections. Specifically, every 25th brain section, with intervals of 250 µm between them, was designated for subsequent immunoenzymatic staining. The protocols for immunostaining were executed in accordance with the methodology documented in prior report [35]. “For the experiment, anti-NeuN antibody was selected because it is an exceptional marker for neurons in the central and peripheral nervous systems at embryonic, juvenile, and adult stages” [205]. Every 25th frozen section was allocated for standard immunoperoxidase labeling utilizing DAB (Dako Liquid DAB + Substrate Chromogen System, K3468, Denmark) as the chromogen, following the methodology outlined in the earlier publication [35]. In summary, selected sections were subjected to an overnight incubation with primary antibodies targeting NeuN (a pan-neuronal marker; Anti-NeuN Antibody, clone A60, MAB377; Merck Millipore, Poland; working dilution 1/1000). Subsequently, these sections underwent a 1 h incubation with secondary antibodies (working dilution 1/1000, ImmPRESS™ Universal Reagent (Vector Laboratories, Newark, United States)). After this procedure, the sections were washed with PBS and incubated for 1 min in a DAB substrate-chromogen solution prepared in accordance with the manufacturer’s instructions. All stained tissues were further rinsed with tap water, rehydrated through a graded alcohol series, cleaned in xylene, and finally mounted using DPX (DPX Mountain for histology; 44581, Sigma Aldrich, Darmstadt, Germany). All staining procedures were conducted at room temperature within humid chambers. The processed sections were subsequently digitized and archived using a PathScan Enabler IV Histology Slide Scanner (Praha, Czech Republic).

#### 4.2.3. Preparation of Brains for Measuring the Content of Inflammatory, Oxidative Stress, and Metabolic Markers

After a 7-day habituation phase, the 24 animals were categorized into the following groups (n = 6 at each group): (1) Control 5-week-old WKYs (juvenile); (2) Control 10-week-old WKYs (maturing); (3) ADHD 5-week-old SHRs (juvenile); and (4) ADHD 10-week-old SHRs (maturing). The animals were anesthetized via an intraperitoneal injection of ketamine (100 mg/kg) and xylazine (10 mg/kg), in adherence to established guidelines for the humane euthanasia of experimental animals. To minimize the influence of ketamine on our data, tissue collection was standardized and performed no later than 10 min after ketamine administration (after cessation of breathing and disappearance of reflexes), based on prior research indicating this window reduces its acute effects on brain biochemistry [206,207]. The same anesthetic protocol was used across all experimental groups to ensure consistency. All brains were collected and cryopreserved in liquid nitrogen (−196 °C) and subsequently stored at −80 °C pending further processing.

### 4.3. Morphometric Analysis of Motor Cortex

#### 4.3.1. Volumetric Analyses

**Figure 6:**
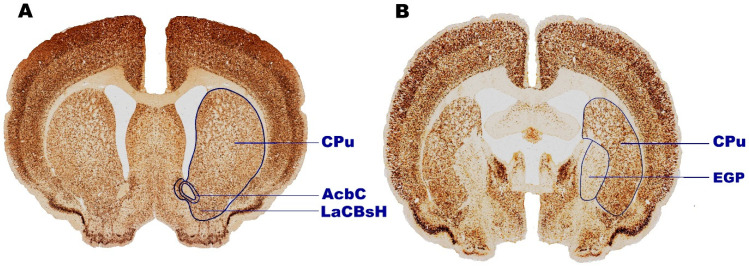
The figure presents stained and digitized (magnification ×5) coronal brain sections of 6-week-old WKYs with the outlined studied areas of the striatum (A: CPu, LaCBsH, and AcbC; and B: CPu and EGP) according to The Rat Brain Atlas [208] ((**A**) bregma 1.20 mm; and (**B**) bregma −0.84 mm).

The boundaries of the distinct striatal areas (Figure 6A,B), including the caudate putamen (CPu), external globus pallidus (EGP), accumbens shell (LaCBsH), and nucleus accumbens core (AcbC), were precisely defined based on coordinates aligning with bregma 2.52 mm to −3.72 mm, referencing the Paxinos and Watson atlas [208]. Additionally, the delineation of the striatal area boundaries followed the criteria proposed by Oorschot [209]. Briefly, volumetric measurements were performed on digitally archived coronal brain sections at a 5.0x magnification using the Fiji image analysis software (FIJI 1.46p, Madison, USA) [210]. Next, the calculation of the total volume of distinct striatal areas was accomplished using the method established by DeVito et al. [211]. According to this mathematical formulation, the total volume of a given structure (Vo) is the sum of the subvolumes within the said structure (Vn). It is noteworthy to mention that the Vn was computed by the multiplication of the 2-D contour area (representing the boundaries of the individual striatal regions) by the inter-section spacing of 250 μm.

#### 4.3.2. The Density Quantification

The quantification of neuronal density within the studied striatal regions (CPu, EGP, LaCBsH, and AcbC) was conducted on stained brain sections. This analysis was carried out using an Olympus BX51 microscope (Olympus GmbH, Hamburg, Germany) equipped with a digital camera (CC-12, Soft Imaging System, Münster, Germany) and the Cell-F software (Cell-F 5.1.0.2108, Olympus, Hamburg, Germany). The count of labeled neurons nuclei was performed in accordance with the method outlined by West and Gundersen [212] at a magnification of 40×, using the 152.86 µm × 114.81 µm fields as the testing frames, considering a section thickness (10 µm). The neuron count obtained for a particular structure was first averaged across all the studied brain sections, and, subsequently, the averages for each developmental stage were calculated and expressed as the number of neurons per cubic millimeter (N/mm^3^).

### 4.4. Assessment of Immune, Oxidative Stress, and Metabolic Markers

A comprehensive explanation for selecting the immune, oxidative stress, and metabolic markers in this study is provided in our previous report [30].

#### Preparation of Test Material and Determination of Immune, Oxidative Stress, and Metabolic Markers/Enzymes

To determine the levels of inflammatory, oxidative stress, and metabolic markers/enzymes in the rats striatum, distinct brain regions were isolated based on the Rat Brain Atlas [208]. Subsequently, the isolated brain parts were homogenized in RIPA buffer at 4 °C. The homogenates were then subjected to centrifugation at 30,000× *g* for 1 h. Following procedure, the resulting tissue supernatants were divided into aliquots and stored at −80 °C. These supernatants were utilized for subsequent measurements. The detailed procedures for quantifying the levels/activity of selected markers are presented in table below (Table 2).

### 4.5. Statistics

The statistical analysis began with preliminary assessments, including normality testing using the Shapiro–Wilk test and homogeneity of variance testing using Levene’s test, which were necessary to confirm the assumptions required for subsequent statistical evaluations. A two-way ANOVA was then conducted with strain and age as independent factors. To obtain reliable, accurate, and properly interpreted statistical results, the Bonferroni post hoc test was subsequently applied. Comparisons between hemispheres were based on ANOVA analysis, which encompassed the entire dataset (for both hemispheres). All statistical procedures were performed using GraphPad Prism 8 (GraphPad Software, La Jolla, CA, USA), with a significance level set at *p* < 0.05. Detailed data for each two-way ANOVA analysis are included in Supplementary Materials.

## 5. Limitations of a Study

Although SHRs are widely used and accepted as a research model for studying changes in striatal regions, such as alterations in structural volume, neuron density, inflammation, and oxidative stress, this animal model has several limitations that may hinder the translation of the findings to human ADHD pathology.

Both rodents and humans exhibit a similar structural organization of the striatum, which is divided into functional subregions. In both species, the striatum is composed of the caudate nucleus and putamen, which are involved in motor control and cognitive functions. The dorsal striatum is primarily associated with motor functions, while the ventral striatum (including the nucleus accumbens) is implicated in reward processing and reinforcement learning [218]. Nevertheless, one of the most notable differences is the anatomical separation of the caudate and putamen in humans, which is marked by the internal capsule. In rodents, these structures are not anatomically distinct and are referred to collectively as the CPu [219].

Moreover, WKYs are genetically related to SHRs, as both strains originate from the same ancestor. This genetic relationship makes WKYs an appropriate control for identifying the behavioral and neurobiological aspects of ADHD in SHRs [220]. WKYs are frequently used as a reference point to evaluate the effects of hypertension and hyperactivity phenotypes observed in SHRs. However, WKYs (aged 7 to 20 weeks) have been identified as a model for depressive behaviors, exhibiting increased immobility in forced swim tests and other indicators of depression [221]. This characteristic can confound results when using WKYs as controls, as any observed behavioral differences may not solely reflect ADHD-related symptoms but could also be influenced by underlying depressive states.

## 6. Conclusions

The results presented in this article regarding striatal changes significantly expand our understanding of ADHD pathophysiology in the SHR animal model. The prepubertal period, corresponding to weeks 5–7 in SHRs (equivalent to 7–10 years of age in humans), appears to be critical for the development of ADHD-related pathologies. During this time, the most substantial morphometric and biochemical changes occur, particularly in the striatum. These changes likely contribute to difficulties in attention and self-regulation, which are hallmark symptoms of ADHD in both SHRs and humans.

Inflammation and oxidative stress, closely interconnected processes, may lead to neuronal damage and neurotransmitter disruptions. Both the existing literature and our findings suggest that inflammation and oxidative stress interfere with normal neurobiological development, causing neuronal damage and morphological changes in brain structures, including the striatum. These areas play a crucial role in attention regulation and behavioral control [222,223]. Additionally, these conditions disrupt the dopaminergic pathways, which are essential for managing attention and impulsivity [34,224]. Studies have demonstrated abnormalities in dopaminergic signaling in individuals with ADHD [225,226] and in SHRs [29]. Such disruptions may impair cortico-striatal circuits, essential for regulating attention and behavior, thus contributing to the core symptoms of ADHD.

Based on the findings presented in this study, several promising research directions can be proposed to advance our understanding and treatment of ADHD. These directions could focus on specific biochemical markers and signaling pathways involved in ADHD pathophysiology, particularly inflammation, oxidative stress, and dopaminergic dysfunction. One potential hypothesis is that targeting inflammation through anti-inflammatory agents or cytokine inhibitors could mitigate ADHD symptoms by reducing neuroinflammation during critical developmental periods. Another avenue involves developing antioxidants or compounds to modulate oxidative stress pathways, potentially restoring dopaminergic signaling and alleviating the behavioral symptoms of ADHD. Investigating how inflammation and oxidative stress impact dopaminergic signaling could lead to targeted interventions that restore normal DA function in ADHD. Lastly, exploring the role of neuroplasticity, precision medicine approaches, and metabolic pathway modulation could offer new therapeutic strategies to address the molecular mechanisms driving ADHD and provide more personalized treatments. In summary, the insights derived from these studies can help bridge the gap between fundamental neurobiological research and clinical applications, offering a pathway toward more precise and effective interventions. This could pave the way for personalized therapeutic approaches addressing the molecular mechanisms underlying ADHD.

## Figures and Tables

**Table 1:** Body weight of SHRs and WKYs at different age stages.

Age (Weeks)	SHRs Body Weight (g)	WKYs Body Weight (g)
4	89.80 ± 1.90	84.30 ± 0.09
5	115.08 ± 6.31	120.08 ± 0.08
6	154.86 ± 6.13	159.28 ± 0.05
7	200.78 ± 5.37	191.13 ± 6.06
8	220.35 ± 10.76	226.95 ± 0.04
9	249.79 ± 9.86	242.67 ± 9.09
10	269.58 ± 7.92	264.95 ± 10.02

**Table 2:** The table outlines comprehensive procedures for quantifying and determining the activity of selected markers.

	Marker/ Enzyme	Determination Method	
**Immune**	IL-1α	The ELISA test plate’s absorbance was quantified using a TECAN Infinite M200 PRO plate reader (Austria) at a wavelength of λ = 450 nm. The outcomes were expressed relative to the amount of protein, presented per milligram.	ELISA Kit for Rat IL-1 alfa (E0071r; EIAab; Wuhan, China)
	IL-1β		ELISA Kit for Rat IL-1 beta (E0563r; EIAab; Wuhan, China)
	IL-6		ELISA Kit for Rat IL-6 (E0079r; EIAab; Wuhan, China)
	mTOR		ELISA kit for Rat Serine/threonine-protein mTOR (ER1520; Wuhan Fine Biotech Co, Ltd.; Wuhan, China)
	TGF-β		TGF beta-1 Multispecies Matched An-tibody Pair, (CHC1683, ThermoFisher Scientific, Waltham, MA, USA)
	AKT-1		ELISA Kit FOR rat RAC-alpha serine/threonine-protein kinase (E0382r; EIAab; Wuhan, China)
	GCsRβ		ELISA kit for rat Glucocorticoid receptor β (E1608r; EIAab; Wuhan, China)
**Oxidative Stress**	MDA	Markers’ levels/activity were assessed following the following:	Weitner et al. procedure [213]
	-SH		Ellman method [214]
	SOD		Tkachenko and Grudniewska protocol [215]
	POD		Kinetic method [216]
	GSR		Tkachenko and Grudniewska method [215]
	GST		Spectrophotometric assay procedure [217]
**Metabolic**	G	Markers’ content/activity was measured employing specific commercial reagent sets	Glucose oxidase reagent set (G7521; Pointe Scientific, Inc.; Poland)
	FrAm		Fructosamine reagent set (F7546; Pointe Scientific, Inc.; Poland)
	Fe		Total iron reagent set (17505; Pointe Scientific, Inc.; Poland)
	LA		Lactate acid reagent set (L7596; Pointe Scientific, Inc.; Poland)
	LDH		Lactate dehydrogenase reagent set (L7572; Pointe Scientific, Inc.; Poland)
	ALT		Alanine transaminase (SGPT) reagent set (A7526; Pointe Scientific, Inc.; Poland)
	AST		Aspartate aminotransferase (SGOT) reagent set; (A7561; Pointe Scientific, Inc.; Poland)

## Data Availability

The data presented in this study are available on request from the corresponding author.

## Supplementary Materials

The following supporting information can be downloaded at: www.mdpi.com/article/10.3390/ijms252413652/s1.
