# Supplementary material for: Alterations in Striatal Architecture and Biochemical Markers’ Levels During Postnatal Development in the Rat Model of an Attention Deficit/Hyperactivity Disorder (ADHD)
Source: Int J Mol Sci. 2024 Dec 20;25(24):13652. doi: 10.3390/ijms252413652 (PMC11680085; doi:10.3390/ijms252413652)
Supplement: Supplementary file 1 [file ijms-25-13652-s001.zip › ijms-3328375-supplementary.pdf]

Supplementary Material : Results of two-way ANOVA for immune (I), oxidative stress (II), metabolic (III) markers, volume of striatal regions (IV), and neuron density in these regions (V)

I

Marker	ANOVA table	F (DFn. DFd)	P value
IL-α	Interaction	$F_{(1.20)} = 3.101$	$p = 0.0935$
	Age	$F_{(1.20)} = 9.394$	$p = 0.0061$
	Strain	$F_{(1.20)} = 5.972$	$p = 0.0239$
IL-β	Interaction	$F_{(1.20)} = 1.641$	$p = 0.2149$
	Age	$F_{(1.20)} = 11.70$	$p = 0.0027$
	Strain	$F_{(1.20)} = 0.1496$	$p = 0.7030$
IL-6	Interaction	$F_{(1.20)} = 2.757$	$p = 0.1124$
	Age	$F_{(1.20)} = 7.596$	$p = 0.0122$
	Strain	$F_{(1.20)} = 6.569$	$p = 0.0186$
m-TOR	Interaction	$F_{(1.20)} = 0.7701$	$p = 0.3906$
	Age	$F_{(1.20)} = 0.1495$	$p = 0.7031$
	Strain	$F_{(1.20)} = 16.66$	$p = 0.0006$
GCsRβ	Interaction	$F_{(1.20)} = 14.36$	$p = 0.0012$
	Age	$F_{(1.20)} = 11.34$	$p = 0.0031$
	Strain	$F_{(1.20)} = 8.595$	$p = 0.0082$
TGF-β	Interaction	$F_{(1.20)} = 0.5126$	$p = 0.4823$
	Age	$F_{(1.20)} = 18.28$	$p = 0.0004$
	Strain	$F_{(1.20)} = 2.035$	$p = 0.1691$
AKT-1	Interaction	$F_{(1.20)} = 0.075$	$p = 0.7866$
	Age	$F_{(1.20)} = 34.46$	$p < 0.0001$
	Strain	$F_{(1.20)} = 16.33$	$p = 0.0006$

II

Marker	ANOVA table	F (DFn. DFd)	P value
MDA	Interaction	$F_{(1.20)} = 103.4$	$p < 0.0001$
	Age	$F_{(1.20)} = 1206$	$p < 0.0001$
	Strain	$F_{(1.20)} = 100.5$	$p < 0.0001$
-SH	Interaction	$F_{(1.20)} = 51.61$	$p = 0.2149$
	Age	$F_{(1.20)} = 1100$	$p = 0.0027$
	Strain	$F_{(1.20)} = 165.6$	$p = 0.7030$
SOD	Interaction	$F_{(1.20)} = 5.250$	$p = 0.0329$
	Age	$F_{(1.20)} = 0.4312$	$p = 0.5189$
	Strain	$F_{(1.20)} = 65.26$	$p < 0.0001$
POD	Interaction	$F_{(1.20)} = 0.7701$	$p = 0.3906$
	Age	$F_{(1.20)} = 0.1495$	$p = 0.7031$
	Strain	$F_{(1.20)} = 16.66$	$p = 0.0006$
GSR	Interaction	$F_{(1.20)} = 0.0699$	$p = 0.7941$
	Age	$F_{(1.20)} = 4.769$	$p = 0.0411$
	Strain	$F_{(1.20)} = 1.732$	$p = 0.2030$
GST	Interaction	$F_{(1.20)} = 2.287$	$p = 0.1461$
	Age	$F_{(1.20)} = 32.03$	$p < 0.0001$
	Strain	$F_{(1.20)} = 0.0977$	$p = 0.7579$

III

Marker	ANOVA table	F (DFn. DFd)	P value
G	Interaction	$F_{(1.20)} = 126.4$	$p < 0.0001$
	Age	$F_{(1.20)} = 422.5$	$p < 0.0001$
	Strain	$F_{(1.20)} = 180.0$	$p < 0.0001$
FrAm	Interaction	$F_{(1.20)} = 44.23$	$p = 0.0001$
	Age	$F_{(1.20)} = 4.599$	$p = 0.0445$
	Strain	$F_{(1.20)} = 20.11$	$p = 0.0002$
Fe	Interaction	$F_{(1.20)} = 8.104$	$p = 0.0100$
	Age	$F_{(1.20)} = 74.78$	$p < 0.0001$
	Strain	$F_{(1.20)} = 7.099$	$p = 0.0149$
LA	Interaction	$F_{(1.20)} = 8.65$	$p = 0.0081$
	Age	$F_{(1.20)} = 311.4$	$p < 0.0001$
	Strain	$F_{(1.20)} = 1.545$	$p = 0.2283$
LDH	Interaction	$F_{(1.20)} = 223.9$	$p < 0.0001$
	Age	$F_{(1.20)} = 117.7$	$p < 0.0001$
	Strain	$F_{(1.20)} = 98.46$	$p < 0.0001$
ALT	Interaction	$F_{(1.20)} = 36.93$	$p < 0.0001$
	Age	$F_{(1.20)} = 67.85$	$p < 0.0001$
	Strain	$F_{(1.20)} = 17.27$	$p = 0.0005$
AST	Interaction	$F_{(1.20)} = 10.72$	$p = 0.0038$
	Age	$F_{(1.20)} = 162.4$	$p < 0.0001$
	Strain	$F_{(1.20)} = 16.42$	$p = 0.0006$

IV

	Region	ANOVA table	F (DFn. DFd)	P value
VOLUME	CPU	Interaction	$F_{(18.132)} = 2.046$	$p = 0.0113$
		Age	$F_{(6.132)} = 139.3$	$p < 0.0001$
		Strain	$F_{(3.132)} = 62.47$	$p < 0.0001$
	EGP	Interaction	$F_{(18.132)} = 1.32$	$p = 0.1870$
		Age	$F_{(6.132)} = 3.955$	$p < 0.0001$
		Strain	$F_{(3.132)} = 34.17$	$p < 0.0001$
	LaCBsH	Interaction	$F_{(18.132)} = 1.655$	$p < 0.0557$
		Age	$F_{(6.132)} = 19.74$	$p < 0.0001$
		Strain	$F_{(3.132)} = 19.93$	$p < 0.0001$
	AcbC	Interaction	$F_{(18.132)} = 3.354$	$p = 0.0003$
		Age	$F_{(6.132)} = 49.49$	$p < 0.0001$
		Strain	$F_{(3.132)} = 6.597$	$p = 0.0003$

V

	Region	ANOVA table	F (DFn. DFd)	P value
NEURON DENSITY	CPU	Interaction	$F_{(18.132)} = 3.47$	$p < 0.0001$
		Age	$F_{(6.132)} = 85.71$	$p < 0.0001$
		Strain	$F_{(3.132)} = 63.28$	$p < 0.0001$
	EGP	Interaction	$F_{(18.132)} = 1.32$	$p = 0.1870$
		Age	$F_{(6.132)} = 7.684$	$p < 0.0001$
		Strain	$F_{(3.132)} = 26.44$	$p < 0.0001$
	LaCBsH	Interaction	$F_{(18.132)} = 4.69$	$p < 0.0001$
		Age	$F_{(6.132)} = 20.65$	$p < 0.0001$
		Strain	$F_{(3.132)} = 19.05$	$p < 0.0001$
	AcbC	Interaction	$F_{(18.132)} = 2.89$	$p = 0.0003$
		Age	$F_{(6.132)} = 120.3$	$p < 0.0001$
		Strain	$F_{(3.132)} = 11.43$	$p < 0.0001$
